# Optimizing alkaline hydrothermal treatment for biomimetic smart metallic orthopedic and dental implants

**DOI:** 10.1007/s10856-024-06794-y

**Published:** 2024-06-19

**Authors:** Hanieh Hadady, Arefin Alam, Indu Khurana, Isha Mutreja, Dhiraj Kumar, Mamilla Ravi Shankar, Rupak Dua

**Affiliations:** 1 Polymer & Material Science Research, Department of Innovation & Technology Research, American Dental Association Science & Research Institute, L.L.C., Gaithersburg, MD USA; 2 Department of Economics and Business, Hampden-Sydney College, Hampden-, Sydney, VA USA; 3 Minnesota Dental Research Center for Biomaterials and Biomechanics, Department of Restorative Sciences, University of Minnesota, Minneapolis, MN USA; 4 Division of Pediatric Dentistry, School of Dentistry, University of Minnesota, Minneapolis, MN USA; 5 Department of Mechanical Engineering, Indian Institute of Technology, Tirupati, AP India; 6 Department of Chemical Engineering, Hampton University, Hampton, VA USA

**Keywords:** Biomimetic, Orthopedic, Dental, Implants, Nanotechnology, Surface modification.

## Abstract

Orthopedic and dental implant failure continues to be a significant concern due to localized bacterial infections. Previous studies have attempted to improve implant surfaces by modifying their texture and roughness or coating them with antibiotics to enhance antibacterial properties for implant longevity. However, these approaches have demonstrated limited effectiveness. In this study, we attempted to engineer the titanium (Ti) alloy surface biomimetically at the nanometer scale, inspired by the cicada wing nanostructure using alkaline hydrothermal treatment (AHT) to simultaneously confer antibacterial properties and support the adhesion and proliferation of mammalian cells. The two modified Ti surfaces were developed using a 4 h and 8 h AHT process in 1 N NaOH at 230 °C, followed by a 2-hour post-calcination at 600 °C. We found that the control plates showed a relatively smooth surface, while the treatment groups (4 h & 8 h AHT) displayed nanoflower structures containing randomly distributed nano-spikes. The results demonstrated a statistically significant decrease in the contact angle of the treatment groups, which increased wettability characteristics. The 8 h AHT group exhibited the highest wettability and significant increase in roughness 0.72 ± 0.08 µm (*P* < 0.05), leading to more osteoblast cell attachment, reduced cytotoxicity effects, and enhanced relative survivability. The alkaline phosphatase activity measured in all different groups indicated that the 8 h AHT group exhibited the highest activity, suggesting that the surface roughness and wettability of the treatment groups may have facilitated cell adhesion and attachment and subsequently increased secretion of extracellular matrix. Overall, the findings indicate that biomimetic nanotextured surfaces created by the AHT process have the potential to be translated as implant coatings to enhance bone regeneration and implant integration.

**Graphical Abstract:**

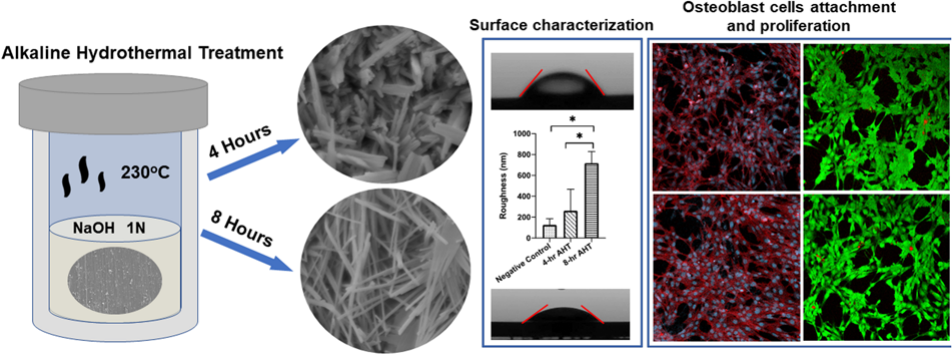

## Introduction

Metallic implants, primarily composed of titanium alloys, have become increasingly prevalent in dental and orthopedic applications over the last decade. In 2010, a staggering 7.2 million Americans underwent total joint replacement surgery, with 2.5 million receiving total hip replacements and 4.7 million undergoing total knee replacements [1]. Similarly, the number of dental implantations increased by 14% per year between 1999 and 2016 [2]. These trends are predicted to inflate exponentially, with projections estimating a 50% increase in hip and knee replacements by 2030 and a 23% increase in dental implants by 2026 [2, 3].

Despite their life-enhancing potential, standard metallic alloy-based implants are associated with numerous challenges. Among these, bone loss surrounding the implant is particularly noteworthy because of the potential bacterial infection in the micro-space due to poor soft tissue attachment. This leads to implant failure, causing revision surgeries that are more complicated and costly, further leading to a socioeconomic impact on the patient and a burden on the healthcare system [4–7]. Standard operating conditions and isolation of patients are often considered during surgical procedures to minimize the risk of bacterial infections [8, 9]. Efforts have also been made to prevent infections using antibiotics as the conventional treatment method [10]. However, this approach has led to the development of antimicrobial-resistant infections, which have become a growing problem in recent years. Chemical modification of the implant surface has also been reported to minimize bacterial attachment, thereby inhibiting biofilm formation [11–13]. Antibiotic coatings such as gentamicin have been immobilized on implant surfaces to kill the bacteria that adhere to the surface [14]. However, the gradual loss of coating while fitting the implant into the bone or during the micromotion of implants can create additional biological concerns, such as aggressive inflammatory response or bone necrosis, further increasing the probability of implant failure [15]. As the demand for metal-based implants continues to grow, it is crucial to address these challenges. There is a critical need to develop implant materials and strategies to promote enhanced integration with the host tissue as well as inherent antibacterial properties to achieve better long-term outcomes.

It is well established that the nanotextured surfaces with variable surface wettability, such as spinules/hair (hydrophobic), nano-grass (hydrophobic/hydrophilic), nanoneedles, spherical nano-cones, nano-hemispheres (hydrophilic), and nanopores (hydrophobic) are known to be toxic for different gram (+) and gram (-) species [16–21]. Besides, these nanotextured topographies are also known to provide cues for mammalian cell stimulation for improved cell attachment to the surface [22, 23]. Furthermore, recent studies have corroborated that naturally existing nanostructured surfaces found on insects and other living species can kill bacterial cells. The potential working mechanism could be rupturing the microbial cell wall through a contact-killing mechanism, thus offering an alternative solution [24, 25]. Therefore, synthesizing such biomimetic nanotextured implant surfaces can potentially reduce implant failure and minimize drug-resistant bacterial infections in healthcare settings.

Various methods have been employed in literature to prepare surfaces on titanium and its alloys, including chemical etching [26–28], sol-gel methods [29], anodization [30], and lithography [31]. Among these, chemical etching in alkali solutions stands out as a facile, high throughput, and cost-effective method capable of producing a titanate layer with randomly oriented nanostructures on the surface of titanium and its alloys [32]. Studies have shown that alkaline hydrothermal treatment (AHT) is being used to modify the surface of metals to enhance their chemical reactivity, adhesion properties, and hydrophilicity for biomedical applications [33, 34]. AHT is a potential approach to create nanostructures on the Ti alloy surface, which can elicit different biological responses depending upon the roughness, wettability, and other surface properties. The AHT is a function of the type of etching agent, its concentration, process time, and temperature [35]. For instance, Diu et al. used NaOH 1 M solution at a temperature of 240 °C for 3 and 8 h for their AHT process. They observed an effective response for their surface against gram-negative bacterial cell lines but not non-motile gram-positive bacteria [36]. Another group employed KOH & NaOH for AHT at 150 °C, resulting in different nanotextures [37]. Additionally, other researchers have investigated the influence of NaOH concentration of 1 M, 5 M and 10 M at 110 °C for 5 h, resulting in the development of varying nanotextures [38]. Even in our previous study, we utilized a 1 N NaOH solution as an etchant at 230 °C, examining two different processing times (4 and 8 h). We successfully created biomimetic nanotexture surfaces based on the cicada wing’s structure on medical grade 5 titanium (Ti) alloy, demonstrating inherent antibacterial properties for both gram-negative and gram-positive [26]. However, their biological responses to mammalian cell lines and surface properties have not been explored in-depth. Despite the considerable body of research on AHT applied to titanium implants, there remains a need for further exploration into the details of the process. Specifically, there is a gap in identifying the optimal processing parameters that influence the final biological and surface properties of the implants.

In this study, we aimed to develop biomimetic Ti surfaces for orthopedic and dental implant applications and evaluate the surface properties and their biological responses using an in vitro model. We hypothesized that the nanospikes created using AHT would allow the larger mammalian cells to adhere and proliferate.

## Material and methods

### Preparation of titanium alloy substrates

A titanium (Ti) grade 5 alloy sheet (McMaster-Carr, Elmhurst, IL, USA) of thickness 0.4 mm was cut into small flat square sections of 10 mm × 10 mm using a band saw. The cut Ti sections were subsequently polished with silicon carbide grinding papers ranging from coarse to very fine grit sizes # 220, 400, 1000, and 1500. During the grinding operation, a steady stream of water was applied to the metallic sections to drive away the ground particles. To maintain uniform grinding and suppress the development of deep grooves, the Ti sections were rotated 90° following each successive grinding. Finally, using 1500-grade grinding paper, mirror-finish samples were obtained. The samples were then ultrasonically washed in acetone for 10 min, ensuring that the samples did not stick to each other. Subsequently, the samples were rinsed with deionized water and air-dried before use for further experimentation.

### Alkaline hydrothermal treatment (AHT)

The small sections of Ti alloys were placed in the acid digestion vessels (Model # 4745, Parr Instrument Company, Moline, IL, USA) for carrying out the AHT for two different reaction times of 4 h and 8 h following our previous study [26]. Briefly, the samples were placed in a polytetrafluoroethylene (PTFE) cup (23 ml capacity) with a cover housed within a metallic body and a screw cap of the acid digestion vessel. The PTFE cup was filled with 10 ml of 1 N sodium hydroxide (NaOH) solution, used as the reactant for alkaline hydrothermal treatment of Ti alloy samples, and was covered with the PTFE cover. The selection of a 1 N NaOH concentration was based on our established methodologies and previous research, ensuring consistency with existing literature [26, 36]. The number of metallic samples per vessel was limited to one to avoid sticking the metal pieces with one another. A corrosion disc (thinner foil) was placed above the PTFE cover along with a blow-off disc. Further, the threaded screw of the cap was lubricated with graphite powders to prevent jamming. The vessel was sealed by hand tightening the screw cap, and then it was turned not more than 1/8 turn using the hook spanner wrench provided by the manufacturer to avoid over-tightening. Once assembled, the digestion vessels were placed in an oven preheated at 230 °C to prepare two groups (4 h and 8 h) of samples. After the reaction, the vessels were removed from the oven and cooled down to room temperature before extracting the Ti sample from the acid digestion vessels. The Ti samples were then thoroughly rinsed with deionized water to remove the unreacted NaOH.

### Post-alkaline hydrothermal treatment and calcination

The AHT processed samples for both groups were placed in a ceramic dish, with the polished surface facing upwards in the oven for 1 h at 230 °C. Subsequently, 0.6 M HCl was poured into the ceramic container and held for five minutes to allow ion exchange and conversion of sodium titanate to TiO_2_ [39, 40]. The samples were then thoroughly rinsed with deionized water to wash off any residual acid. Finally, the samples were transferred to a furnace maintained at 600 °C for 2 h for calcination to oxidize the samples completely. After calcination, the samples were stored, sterilized with 70% ethanol, and rinsed with phosphate buffer solution before subsequent experimentations.

In sum, three (3) groups of Ti samples were prepared: 1) Group 1: Negative Control (Ti samples with no AHT); Group 2: 4 h AHT (Ti samples subjected to 4 h of AHT at 230 °C); 3) Group 3: 8 h AHT (Ti samples subjected to 8 h of AHT at 230 °C); 4) Tissue culture polystyrene (TCPS) plates characterized by a roughness ranging from 0.1 to 5 nm [41], serve as the positive control, with cells cultured in a well plate.

### Surface topology

The surface topography of the Ti specimens for the three groups was analyzed using a field emission scanning electron microscope (JEOL 6330 F, JEOL Ltd., Akishima-Shi, Tokyo, Japan). The specimens (*n* = 3 per group) were first sputter coated with a 10 nm layer of platinum before observing them under SEM at a voltage of 5 kV and a working distance of 15–16 mm. Further, the length and % area of the nanospikes was calculated from the observed images using ImageJ. The examination was conducted on randomly selected regions to provide representative images of the unique topology of the Ti plates in different groups.

### Wettability

The wettability of different groups was determined by measuring the contact angles under ambient conditions (Temperature 20 °C, 40% humidity) using a sessile drop method with a goniometer (Ossila Limited, Sheffield, United Kingdom), as conducted previously [42]. Deionized water was employed as the solvent to carry out the test. A drop of water was placed on the surface of samples (*n* = 3/group), which was recorded for 10 s using a high-resolution camera. Three measurements were recorded at different locations on each specimen with sufficient spacing to prevent any interference from previous tests. The contact angle θ, which is the angle between the solid surface and the liquid interface, was measured using the measurement software provided with the instrument. The average contact angle of each group was determined and recorded.

### Roughness

A Dektak XTL Stylus Profiler (Brucker, Billerica, MA) was employed to measure the surface roughness of all three groups of titanium samples. Three samples from each group were randomly selected, and the stylus of the profiler was moved three times for a 3 mm distance at a speed of 0.4 mm/sec and a force of 10 mg on different areas of each sample to provide a raw roughness value. Utilizing custom-written MATLAB code, the average values of the raw roughness for each group were recorded and compared.

### In-vitro cell studies

Ti samples were randomly selected from each group. In addition to using 70% ethanol, the samples were sterilized with ultraviolet light for one hour in the cell culture hood before performing cell culture experiments. The human osteoblast cell line (hFOB 1.19, ATCC, Manassas, VA, USA) were cultured until passage 2 (P2) in a complete growth medium at 34 °C, and 5% CO_2_ in a humidified atmosphere, as previously reported [43]. Briefly, the cells were thawed and transferred into the complete media comprised of the base medium (Catalog # 11039021, Gibco, Fischer Scientific, Hanover Park, IL), which is a 1:1 mixture of DMEM/F-12 with 2.5 mM L-glutamine that was further supplemented with 10% fetal bovine (Catalog # 16000044, Fischer Scientific), 1% Penstrep (Catalog# 15140122, Fischer Scientific) to 1% and 0.3 mg/ml of G418 disulphate solution (Catalog# G8168, Sigma Aldrich Inc.). Cultivating osteoblasts at 34 °C rather than the more common 37 °C aligns closer with in vivo bone tissue conditions, ensuring experiments yield more applicable and meaningful outcomes. This temperature adjustment has been shown to not only bolster osteoblast differentiation and function but also to stimulate the expression of bone-specific genes and the synthesis of extracellular matrix components, effectively replicating the natural bone formation process more accurately [44, 45].

#### Cell Attachment and Adhesion (Cytoskeleton Imaging—DAPI, Phalloidin)

To explore cell adhesion and their morphology on different groups of Ti substrates, staining procedures were implemented, involving counterstaining with DAPI (stains nuclei) and Phalloidin-iFluor 594 Reagent (ab150117) (stains cytoskeleton) at a dilution of 1:1000 in PBS containing 1% BSA. After a 48-hour incubation, cells on different Ti substrates were washed twice with PBS and then moved to fresh wells in a new 48-well plate. Following this, hFOBs were fixed with a 4% paraformaldehyde solution (PFA) for 10 min, permeabilized with Triton X-100 (0.1 v/v%) for 5 min and stained with fluorescein phalloidin for 1 h. Cell nuclei were further labeled using DAPI (Invitrogen). Fluorescence imaging of all samples was performed using a confocal laser-scanning microscope equipped with a 10x objective lens (Zeiss, LSM800). The area of cell adhesion was analyzed using ImageJ (Rasband, W.S., ImageJ, U.S. National Institutes of Health, Bethesda, Maryland, USA) with the respective F-actin (red) and cell nuclei (blue) channels as previously reported [46]. The average cell area was calculated using the formula:

$${Average}\,{cell}\,{area}=\frac{{Total}\,{cell}\,{area}}{{Number}\,{of}\,{cells}}$$

#### Cell Viability

To assess cell viability on Ti samples (*n* = 3/group), a live-dead assay employing Calcein AM/Ethidium homodimer was performed. Ti samples from each group were placed in a 48-well plate and seeded with the cells at a density of 2.5 × 10^5^ cells/ ml. Following a 48 h incubation period, the samples were washed and transferred to the new wells before initiating the assay with the Live/Dead cell imaging kit 488/570 (ThermoFisher Scientific) in accordance with the manufacturer’s instructions. In brief, the samples underwent staining with Calcein AM and Ethidium homodimer in PBS solution for 15 min in a dark environment at room temperature. Subsequently, the samples were rinsed twice with phosphate-buffered saline to eliminate the background fluorescence and visualized using the Zeiss confocal laser-scanning microscope at excitation and emission wavelengths of 488 nm and 570 nm, respectively.

#### Cytotoxicity and proliferation assessment

The cytotoxicity of osteoblastic hFOB 1.19 cells exposed to the three different surfaces (*n* = 3/group) was evaluated using sulforhodamine B (SRB) assays. Cells were seeded at a density of 1 × 10^5^ cells/ml and incubated in a standard cell culture environment for osteoblasts (5% CO_2_ and 34 °C) for 1, 3, and 7 days. The SRB assay was conducted using the CytoScanTM SRB Cytotoxicity Assay Kit (Catalog # 89028079, Fischer Scientific) as per a previously published method [47, 48], and the cellular viability was quantified by the colorimetric measurement of the cellular protein. The process involved fixing the cells on the sample surface, washing the fixing reagent, staining the protein within the viable cells with SRB dye, rinsing the cells to remove the unbound dye, and extracting the SRB-bound protein solution for quantification. The absorbance of the collected solutions was measured at 565 nm using a microplate reader, and cellular viability was reported as “Relative Survival” based on the absorbance values. The results were normalized such that the average absorbance of TCPS positive control group was equal to one. The background absorbance was subtracted, and each absorbance value was divided by the average absorbance value of the control group (data represented as mean ± standard error (SE)). A higher absorbance value indicates lower cell toxicity due to a more significant number of viable cells that can be correlated to cell proliferation.

#### Alkaline phosphatase activity

The quantification of cell differentiation for each group of Ti plates/substrates was conducted by measuring the Alkaline Phosphatase (ALP) activity of the control and exposed cells (*n* = 3/group) using a fluorometric assay (Catalog # 89028079, Fischer Scientific) as previously conducted [49]. The cells were plated on different substrates in a 24-well plate at a cell density of 1 × 10^5^ cells/ml, and ALP activity was measured at respective time points. At each time point, cells were trypsinized from each sample, and the cell pellet obtained was homogenized in 100 µl of assay buffer. Then, 30 µl of cell lysate from each sample in triplicates was diluted with the assay buffer to a total volume of 110 µl and mixed with 20 µl of 0.5 mM of the non-fluorescent substrate 4 methylumbelliferyl phosphate (MU) and incubated for 30 min at room temperature in the dark. Background controls were prepared similarly using the assay buffer, which was mixed with the non-fluorescent substrate and 20 µl of stop solution. The reaction was stopped by adding 20 µl of stop solution (except for the background samples) and shaking gently. The fluorescence intensity was measured using a fluorescence microplate reader at Ex/Em 360/400 nm, with values corrected by subtracting the background control samples. The amount of 4-MU generated was calculated using a standard curve (prepared using the manufacturer’s instructions). ALP activity was calculated in mU/ml using the formula ALP activity = A/V/T (where A = amount of 4-MU generated, V = volume of samples in the assay well, and T = reaction time). To normalize the ALP activity of different groups at each time point, the ALP activity of each group was divided by the average ALP activity of the control group at the corresponding time point.

### Statistics

The results for wettability, roughness, cytotoxicity, and ALP activity were expressed as mean ± standard deviation (SD) unless specified something else. Statistical analysis was done using SPSS (IBM, version 27). A one-way ANOVA with Tukey’s post-hoc test was performed to compare the means of different groups at a 5% level of significance unless stated otherwise.

## Results

### Surface topology

**Fig. 1:**
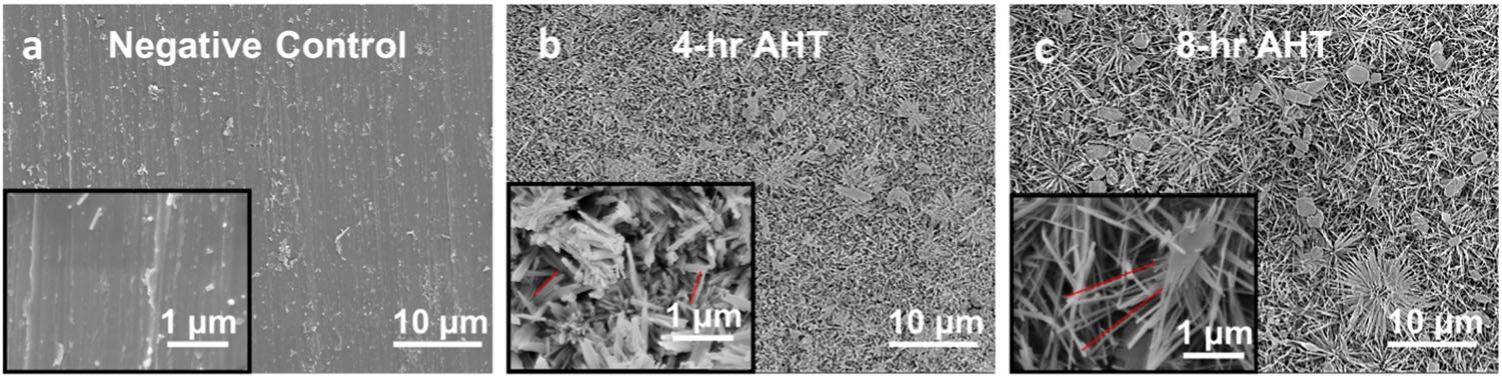
SEM micrographs showing the surface topology of Ti specimens for different groups. **a** Nega-tive Control group exhibited relatively smooth surface with little artifacts while nanoflowers containing randomly distributed nano-spike structures in the range of 250–350 nm and 1000–1250 nm were ob-served in 4 h and 8 h AHT specimens respectively. Panel (**b**, **c**) as indicated by the red lines. Insets provide a closer, magnified view of the images

SEM imaging revealed different surface topographies for different groups as shown in Fig. 1. The negative control group (Group 1) exhibited a relatively smooth surface with minimal artifacts (Fig. 1a), whereas the surfaces of Group 2 (4 h) and Group 3 (8 h) AHT specimens displayed nanoflower structures containing randomly distributed nano-spikes (Fig. 1b, c) similar to nanostructures reported previously for hydrothermal etching of Ti [27]. We observed that the average height of the spikes ranged from 250 to 350 nm for the 4 h AHT group, whereas it measured in the range of 1000–1250 nm for the 8 h AHT group, as highlighted by the red lines (Fig. 1b, c). Additionally, the calculated spatial area of the nanospikes from both the 4 h and 8 h AHT images was approximately 37%.

Fig. 1 SEM micrographs showing the surface topology of Ti specimens for different groups. Fig. 1a: Negative Control group exhibited relatively smooth surface with little artifacts while nanoflowers containing randomly distributed nano-spike structures in the range of 250–350 nm and 1000–1250 nm were observed in 4 h and 8 h AHT specimens respectively (Fig. 1b, c) as indicated by the red lines. Insets provide a closer, magnified view of the images.

### Wettability

**Fig. 2:**
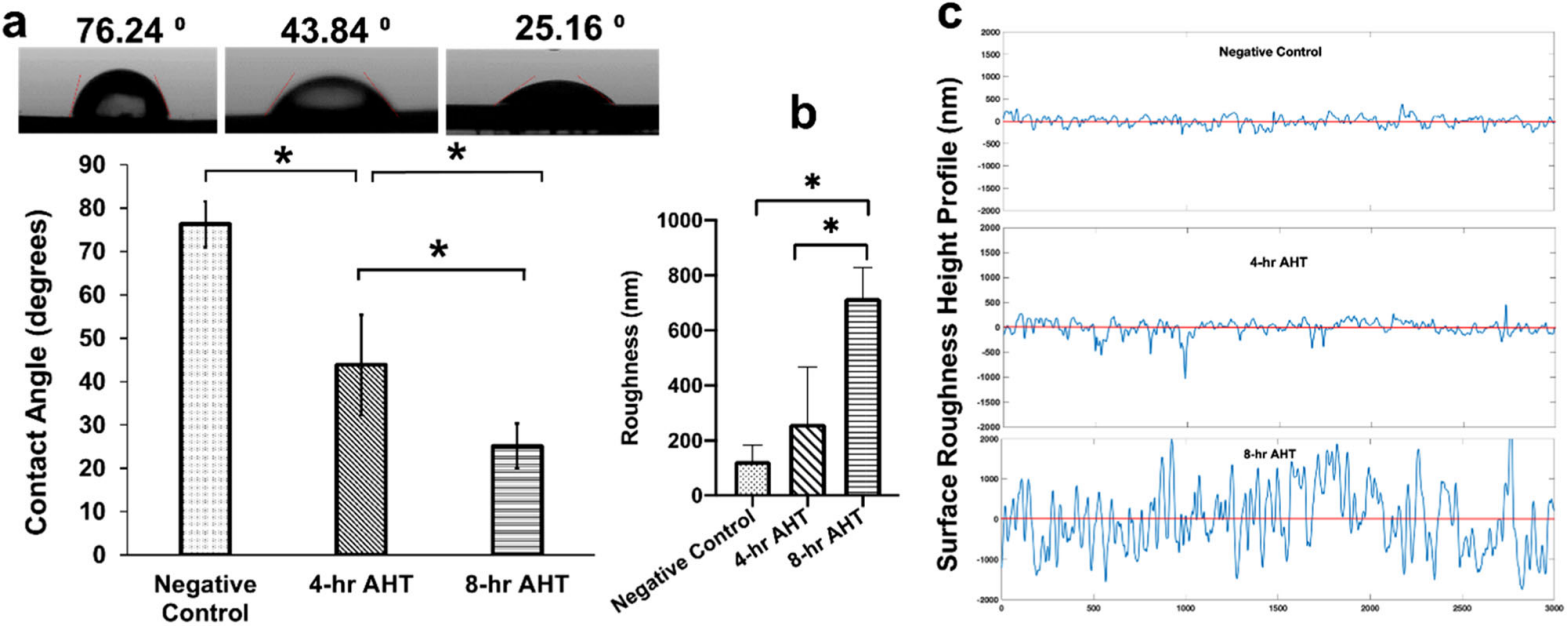
**a** Contact Angle measurement using Goniometer for different groups. **b** Surface Roughness measured using Dektak XTL Stylus Profiler. **c** Surface roughness height profile measured by randomly selecting one part of the surface from each group. We found a significant difference between the contact angles and roughness values of the negative control and the treatment groups, Group 1: Negative control; Group 2: 4 h AHT; Group 3: 8 h AHT, The “*” indicates that the difference between the groups was statistically significant (*P* < 0.05), mean ± SD, *n* = 3

The average contact angle measured for the negative control group surface was 76.24 ± 5.29°. There was a statistically significant decrease (*P* = 0.004 and *P* = 0.000) in the contact angle for both the treatment groups (4 h and 8 h AHT) compared to the negative control group respectively. The average contact angle was 43.84 ± 11.55° and 25.16 ± 5.22° for the 4 h and 8 h AHT groups, respectively. Furthermore, there was a statistically significant difference (*P* = 0.008) between the contact angles for the 4 h and 8-hr AHT groups (Fig. 2a).

### Roughness

We observed a relatively smooth surface for the negative control group, with a roughness value of 130 ± 30 nm, which was statistically lower than the values of the treatment groups. The average roughness value for the 4 h AHT group was 260 ± 100 nm, higher than the negative control group value, while for the 8-hr AHT group, the average roughness value was 720 ± 80 nm, significantly higher than both the negative control group (*P* = 0.015) and the 4 h AHT group (*P* = 0.039) (Fig. 2b, c).

### In-vitro cell studies

#### Cell attachment & adhesion

**Fig. 3:**
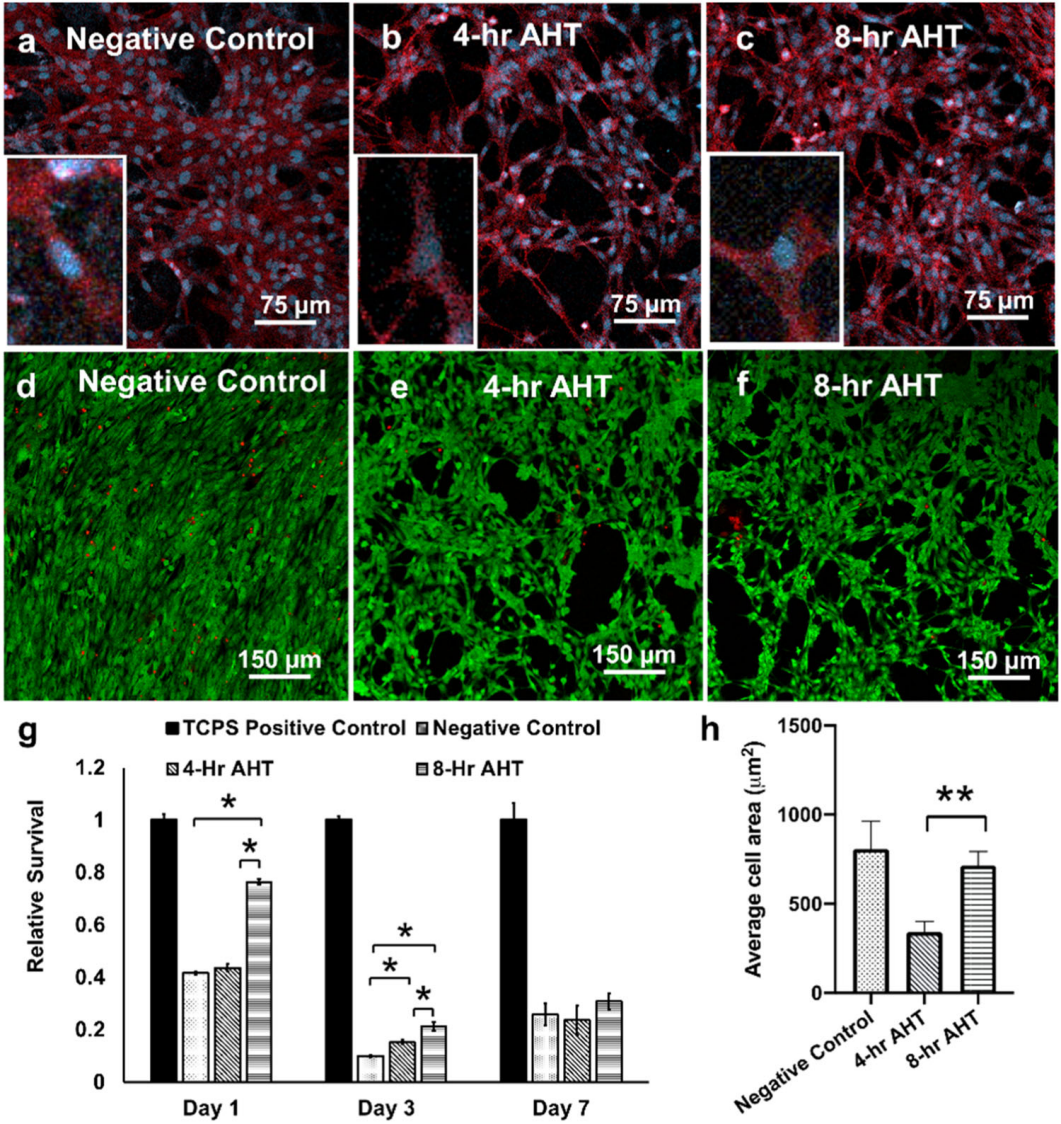
**a**–**c** Cytoskeleton (red fluorescence) and nuclei (blue fluorescence) staining showing the cell adhesion to the surface after 48 h of cell culture with different surface topologies. The surfaces appeared to have a different response to the cell attachment. Insets provide a closer, magnified view of the cells morphology and expansion. **d**–**f** Viability of cells in direct contact with the treated surfaced using confocal microscope (green fluorescence—viable cells, red fluorescence—dead cells) **g** Cell toxicity of osteoblastic cells normalized to TCPS positive control group on different surfaces showed a higher relative survivability of osteoblastic cells in the 8 h AHT group compared with the negative control and 4 h AHT groups on over a period of 7 days. The “*” indicates that the difference between the groups was statistically significant (*P* < 0.05). **h** Average cell area calculated from cytoskeleton staining, The “**” indicates that the difference between the groups was statistically significant difference (*P* < 0.1), Group 1: Negative control; Group 2: 4 h AHT; Group 3: 8 h AHT, mean ± SD, *n* = 3

The cytoskeleton and nuclei of hFOB cells cultured on different Ti substrates (Negative Control, 4 h AHT, and 8 h AHT) for 48 h are illustrated in Fig. 3a–c. While hFOB cells on the negative control surface displayed elongated cell bodies, while those incubated on 4 h AHT and 8 h AHT surfaces exhibited spindle-like morphology with thinner and more elaborated cell extensions. Further, we found that the average cell area for the 8 h AHT group was statistically higher compared to 4 h AHT (*P* = 0.082) at 90% confidence level (Fig. 3h).

#### Cell viability

The viability of the hFOB cells on the three different Ti substrates was observed after 48 h of culture. As shown in the representative images of the live-dead assay fluorescence images (Fig. 3d–f) the hFOB cells were found to be viable in all three Ti groups. It’s important to note that the overlapping arrangement of cells hindered our ability to quantify cell viability accurately.

#### Cytotoxicity and proliferation assessment

Osteoblastic cells exhibited successful adherence to the substrate surfaces, initiating a rapid proliferation phase as early as day 1, with a significant difference observed for the 8 h AHT group compared to both the negative control (*P* < 0.05) and the 4 h AHT group (*P* < 0.05). This proliferation trend continued over the 3-day incubation period, demonstrating a significant increase (*P* = 0.045 and *P* = 0.000) in the relative survival for both treatment groups (4 h and 8 h AHT) compared to the negative control group, respectively. Additionally, a statistically significant difference (*P* = 0.017) was observed in the relative survival between the 4 h and 8 h AHT groups. However, by Day 7, no significant difference in relative cell survival was observed between the negative control group and the treatment groups (Fig. 3g).

#### Alkaline phosphatase (ALP) assay

**Fig. 4:**
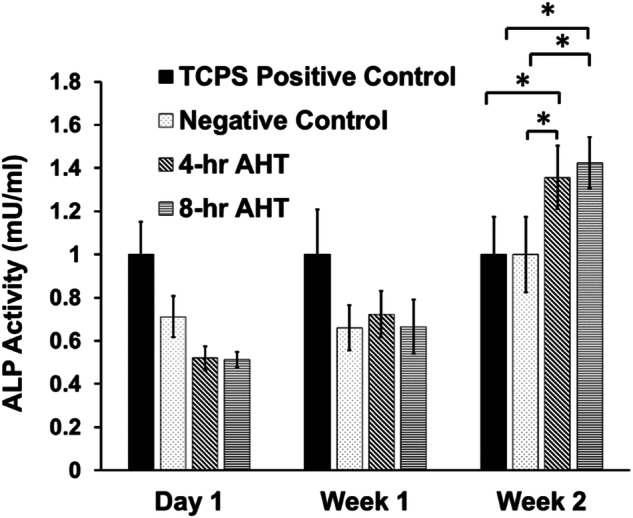
ALP activity in different groups using the fluorometric method over a period of 14 days. A statistical increase in ALP activity for cells in the 4 h and 8 h AHT groups compared to cells in TCPS positive and negative control groups was observed at week 2. The “*” indicates that the difference between the groups was statistically significant (*P* < 0.05), mean ± SD, *n* = 3

In this study, we utilized an ALP assay to evaluate the impact of different Ti nanotextured surfaces on the osteoblastic differentiation potential, without the addition of differentiation growth factors. The results, expressed as the mean ± standard deviation (U/ml), are presented in Fig. 4. After two weeks, the 8 h group samples exhibited the highest alkaline activity (1.42 ± 0.12 U/ml) among all three titanium surfaces tested. The 4 h group demonstrated an ALP activity of 1.36 ± 0.15 U/ml. The flat polished Ti control exhibited an activity of 1.00 ± 0.17 U/ml. Notably, the AHT-treated groups showed a statistically significant increase (*P* = 0.000) in ALP activity compared to the TCPS positive and Negative control groups. However, no significant difference (*P* = 0.821) was observed in the ALP activity of cells on the 4 h and 8 h treated group surfaces.

## Discussion

We observed that the 4 h and 8 h AHT of Ti-grade 5 alloys specimen resulted in nano-scale features on the surface, facilitating the adhesion and proliferation of mammalian cells. The nano-scale features, resembling nanoflowers (Fig. 1a), developed on Ti surfaces through AHT had an average spike height of approximately 250–350 nm for the 4 h group and 1000–1250 nm for the 8 hr group. Both groups had similar spatial coverage, with the measured percent area being approximately 37%. This indicates a consistent distribution of the spikes over the unit area for both treatment groups. However, the length of these nanospikes significantly changed the surface topology, contact angle, and surface roughness of the treatment groups.

We then assessed surface wettability by measuring the contact angles of the treatment groups and compared our findings against those derived from the negative control group. A water contact angle is a standard to define whether the surface is hydrophilic (angle < 90°) or hydrophobic (angle > 90°) [50]. Our results revealed a noteworthy and statistically lower (*P* < 0.05) contact angle values of the treatment groups when compared to the flat-polished negative control group. The contact angles formed on the 4 h and 8 h AHT groups were 43.84 ± 11.55° and 25.16 ± 5.22° respectively, compared to the 76.24 ± 5.29° for the untreated smooth surface of the negative control group. This reduction implies an enhancement in wettability characteristics, indicative of a more hydrophilic surface.

Furthermore, our observations indicated a correlation between the duration of AHT and the increase in wettability. Specifically, surfaces subjected to an 8-hr AHT exhibited contact angle values that were statistically lower (*P* = 0.008) than those of the 4 h AHT groups, signifying a higher degree of wettability. This implies that the duration of AHT plays a pivotal role in influencing the wettability characteristics of the treated surfaces.

With more time for the AHT, we found a statistically significant increase (*P* < 0.05) in the roughness for the 8-hr AHT groups compared with negative control and 4-hr AHT groups. This may be due to the formation of nanospikes on the Ti alloy surface during the AHT, which involves a series of chemical reactions and physical processes. In brief, during the AHT process, the sodium hydroxide (NaOH) solution first reacts with the Ti alloy surface, leading to the formation of the titanium oxide (TiO_2_) layer. Then, the TiO_2_ layer undergoes a dissolution-precipitation process, where the dissolution of TiO_2_ is followed by the precipitation of TiO_2_ nanoparticles on the surface. Finally, the TiO_2_ nanoparticles coalesce to form nanospikes, which are vertically oriented structures with a high aspect ratio. The size and shape of the nanospikes depend on the AHT processing parameters, such as the temperature, pressure, and duration of treatment, as well as the composition of the Ti alloy and NaOH concentration [28, 51–54]. Therefore, in our case, 8-hr AHT formed longer nanospikes, which might have increased the roughness of this group compared with the 4-hr AHT group that had short nanospikes.

It has been established that cell adhesion depends on surface wettability and roughness, which can dictate cell-cell and cell-surface interactions and cell behavior [55, 56]. The enhanced wettability of the treated surfaces, achieved through alkaline hydrothermal treatment (AHT), is likely to improve the interaction with culture media in a manner similar to its interaction with DI water. This enhanced surface-media interaction is anticipated to facilitate a more favorable environment for competitive adsorption of fibronectin present in the culture medium leading to increased cell attachment and proliferation [57]. As shown previously, the cell attachment process progresses through three distinct stages marked by an increase in the number of attached cells with time (stage I), flattening of cells (stage II), until they reach the stable stage of elongation of cells (stage III) on the surface [58]. In our study, we observed the cells’ morphology starting from day 1 for all groups, at which point all the cells had reached a stable phase of attachment and organization.

Previous studies have indicated that surfaces with varying roughness can induce distinct phenotypes, affecting morphology, size, and cytoskeletal organization, with reported optimum surface roughness ranging from 0.13 to 4 µm [59–62]. In our study, the 8 h AHT group exhibited higher roughness of 0.72 ± 0.08 µm. This resulted in increased cell adhesion on this surface and was confirmed from our cell adhesion study where we found that the 8-hr AHT group had significantly higher value of average cell area compared to the 4-hr group (Fig. 3h). The observed spreading of cells on these surfaces is attributed to interactions with the nanostructures.

Moreover, diverse cell morphologies, such as elongated cell bodies and spindle-like shapes, were observed on the flat polished surface (negative control group) and in our treatment groups (4 h and 8 h AHT) respectively. This observation is consistent with findings from previous studies on osteoblast morphology, where similar phenotypes were documented on both smooth and rough surfaces [37, 63].

Our cell toxicity assay (Fig. 3g) suggested that the 8 h AHT group exhibited the lowest cytotoxicity among the tested surfaces before reaching the steady state phase on day 7, as evidenced by the higher relative survivability of the osteoblastic cells. These cytotoxic results can be linked to cell adhesion and proliferation, indicating that the 8 h AHT group might have allowed for more cell attachment than the 4 h AHT group. These findings were further confirmed through cytoskeleton staining and Live/Dead assay after 48 h hFOB culture on different Ti substrates. Indeed, the two distinct topographies primarily varied in the size and shape of their surface structures. This difference in surface structures also influences their mechanobactericidal activity as shown previously in our study [26].

The alkaline phosphatase (ALP) activity, indicative of osteoblastic activity and differentiation and providing valuable insights into the bone formation potential of the surface was measured for four groups: TCPS positive control, negative control, 4 h AHT, and 8 h AHT groups. The results indicated that the 8-hr AHT group exhibited the highest ALP activity, which was significantly higher (*P* < 0.05) than that of the TCPS positive and negative control groups. These findings correlate with the observed morphology in cytoskeleton staining, indicating that the increased cell attachment area or improved cell-surface interaction might have contributed to an increase in ALP activity levels. This increase, in turn, could potentially support enhanced matrix mineralization. Similar effects were observed in previous studies, where higher roughness levels ensured better tissue adhesion and integration between the implant and bone, subsequently positively impacting the healing time after implantation [64, 65]. Moreover, osteogenic differentiation is recognized as a multistep process characterized by a peak in ALP activity, followed by a subsequent decline during the mineralization phase [66]. The 8 h AHT sample results suggest that the osteogenic differentiation process was still ongoing after a period of 2 weeks.

In our previous study, even though we followed a similar methodology for preparing treatment group 4 h AHT and 8 h AHT, our primary emphasis was on assessing the antibacterial properties of those modified surfaces. The results in that prior study revealed significant bactericidal properties for Gram-positive and Gram-negative bacteria and motile/non-motile bacterial strains [26]. When considering our prior findings in conjunction with the current results of this study, it overall suggests that the biomimetic nanospikes inspired by the cicada wing structure, created by AHT, not only enhance the adhesion and proliferation of bone cells on biomimetic surfaces—particularly the 8 h AHT surfaces—leading to enhanced bone mineralization, but may also potentially provide inherent antibacterial properties simultaneously. These findings have important implications for the advancement of new implant materials that can promote osseointegration and improve the long-term success of orthopedic and dental implants while preventing bacterial infections. In particular, biomimetic surfaces created by AHT may have potential applications in developing implant coatings or surface modifications that can enhance bone regeneration and integration and could simultaneously prevent bacterial infection.

Although our results are encouraging for the development of smart surfaces for metallic implants that can promote the adherence, proliferation, and differentiation capability of osteoblastic cells to enhance osseointegration and possess an inherent ability to eradicate bacteria on contact, the long-term stability of these nanostructures are yet to be assessed. Additionally, all our investigations were conducted in vitro, highlighting the need to evaluate further their clinical efficacy in ex vivo and in vivo models. Furthermore, all our investigations were conducted in vitro, emphasizing the need to evaluate their clinical efficacy further using ex vivo and in vivo models. Additionally, while we chose the hFOB 1.19 cell line for its relevance to osteoblast behavior and widespread use in bone-related research, it may not fully represent the diversity of cell responses in clinical scenarios. To address this limitation and better mimic the complex cellular environment encountered clinically, future studies should incorporate primary cells derived from patient samples or co-culture systems involving multiple cell types to provide a more comprehensive understanding.

## Conclusions

In conclusion, our study highlights the efficacy of alkaline hydrothermal treatment in creating biomimetic surfaces with nanospikes on Ti alloy surfaces. Our findings demonstrate that both the 4-hour and 8-hour AHT of Ti-grade 5 alloy specimens resulted in nano-scale features on the surface, which significantly enhanced the adhesion and proliferation of mammalian cells. Specifically, the longer duration of AHT led to the formation of longer nanospikes, increased surface roughness, and improved wettability, all of which contributed to enhanced osteoblast adhesion and proliferation. The observed effects on cell adhesion and proliferation suggest that these nanotextured surfaces hold promise for improving clinical outcomes, such as faster osseointegration and reduced implant failure rates. Furthermore, the bactericidal properties of these surfaces further enhance their potential for clinical application, as they may help reduce the risk of post-operative infections. However, it is important to note that further research is necessary to fully understand the long-term stability of surface nanostructures and their clinical effectiveness. This underscores the importance of continued investigation into the potential benefits and challenges associated with implementing this innovative approach in orthopedic and dental implant.

## Data Availability

The datasets generated during and/or analyzed during the current study are available from the corresponding author upon reasonable request.

## References

[1] Kremers HM, Larson DR, Crowson CS. et al. J Bone Joint Surg. 2015;97:1386 26333733 10.2106/JBJS.N.01141 PMC4551172

[2] Elani H, Starr J, Da Silva J, Gallucci G. J Dent Res. 2018;97:1424–30. 30075090 10.1177/0022034518792567 PMC6854267

[3] Etkin C, Springer B. Arthroplast Today. 2017;3:67–9. 10.1016/j.artd.2017.02.002 PMC5485224 28695176

[4] Sansone V, Pagani D, Melato M. Clin Case Miner Bone Metab. 2013;10:34 10.11138/ccmbm/2013.10.1.034 PMC3710008 23858309

[5] Muratore M, Quarta E, Quarta L, Calcagnile F, Grimaldi A, Orgiani MA, et al. Clin cases Miner bone Metab. 2012;9:50–5. 22783337 PMC3392667

[6] Liaw K, Delfini RH, Abrahams JJ. Seminars in Ultrasound, CT and MRI. 2015;36:424–33. 10.1053/j.sult.2015.09.007 26589696

[7] Tao B, Lin C, He Y, et al. Chem Eng J. 2021;423:130176.

[8] Song Z, Borgwardt L, Høiby N, Wu H, Sørensen, A Borgwardt A. Orthopedic Rev. 2013;5:65–71. 10.4081/or.2013.e14 PMC3718238 23888204

[9] Strachan CJL. J Hospital Infect. 1995;30:54–63. 10.1016/0195-6701(95)90006-3

[10] Romanò CL, Scarponi S, Gallazzi E, Romanò D, Drago L. J Orthop Surg Res. 2015;10:157. 26429342 10.1186/s13018-015-0294-5 PMC4591707

[11] Chouirfa H, Bouloussa H, Migonney V, Falentin-Daudré C. Acta Biomaterialia. 2019;83:37–54. 30541702 10.1016/j.actbio.2018.10.036

[12] Bazaka K, Jacob MV, Crawford RJ, Ivanova EP. Appl Microbiol Biotechnol. 2012;95:299–311. 22618687 10.1007/s00253-012-4144-7

[13] Yoshinari M, Kato T, Matsuzaka K, Hayakawa T, Shiba K. Biofouling. 2010;26:103–10. 20390560 10.1080/08927010903216572

[14] Fuchs T, Stange R, Schmidmaier G, Raschke MJ. Arch Orthop Trauma Surg. 2011;131:1419–25. 21617934 10.1007/s00402-011-1321-6 PMC3175046

[15] Zhang B, Myers D, Wallace G, Brandt M, Choong P. Int J Mol Sci. 2014;15:11878–921. 25000263 10.3390/ijms150711878 PMC4139820

[16] Ivanova EP, Hasan J, Webb HK, Gervinskas G, Juodkazis S, Truong VK, et al. Nat Commun. 2013;4:2838. 24281410 10.1038/ncomms3838 PMC3868328

[17] Hasan J, Raj S, Yadav L, Chatterjee K. RSC Adv. 2015;5:44953–9. 29075481 10.1039/C5RA05206H PMC5654505

[18] Fisher LE, Yang Y, Yuen M-F, Zhang W, Nobbs AH, Su B. Biointerphases. 2016;11:011014. 26992656 10.1116/1.4944062

[19] May PW, Clegg M, Silva TA, Zanin H, Fatibello-Filho O, Celorrio V, et al. J Mater Chem B. 2016;4:5737–46. 32263865 10.1039/c6tb01774f

[20] Hizal F, Zhuk I, Sukhishvili S, Busscher HJ, van der Mei HC, Choi C-H. ACS Appl Mater interfaces. 2015;7:20304–13. 26305913 10.1021/acsami.5b05947

[21] Bhadra CM, Khanh Truong V, Pham VT, et al. Sci Rep. 2015;5:16817. 26576662 10.1038/srep16817 PMC4649496

[22] Holban AM, Gestal MC, Grumezescu AM. Curr Med. Chem. 2014;21:3375–82. 24606502 10.2174/0929867321666140304103810

[23] Stevens MM, George JH. Science. 2005;310:1135–8. 16293749 10.1126/science.1106587

[24] Tripathy A, Sen P, Su B, Briscoe WH. Adv colloid interface Sci. 2017;248:85–104. 28780961 10.1016/j.cis.2017.07.030 PMC6643001

[25] Jaggessar A, Shahali H, Mathew A, Yarlagadda PK. J Nanobiotechnol. 2017;15:1. 10.1186/s12951-017-0306-1 PMC5625685 28969628

[26] Elliott DT, Wiggins RJ, Dua R. J Biomed Mater Res Part B: Appl Biomater. 2021;109:973–81. 10.1002/jbm.b.34762 33241668

[27] Vishnu J, Manivasagam VK, Gopal V, Garcia CB, Hameed P, Manivasagam G, et al. Nanomed: Nanotechnol, Biol Med. 2019;20:102016. 10.1016/j.nano.2019.102016 31158499

[28] Zavala MÁL, Morales SAL, Ávila-Santos M. Heliyon. 2017;3:e00456. 29264415 10.1016/j.heliyon.2017.e00456 PMC5727554

[29] Chang Y-C, Lee C-Y, Chiu H-T. ACS Appl Mater interfaces. 2014;6:31–5. 24341683 10.1021/am405149a

[30] Sjöström T, McNamara LE, Yang L, Dalby MJ, Su B. ACS Appl Mater Interfaces. 2012;4:6354–61. 23138392 10.1021/am301987e

[31] Dickson MN, Liang EI, Rodriguez LA, Vollereaux N, Yee AF. Biointerphases. 2015;10:021010. 10.1116/1.4922157 PMC4474951 26077558

[32] Guo Z, Jiang N, Chen C, Zhu S, Zhang L, Li Y. Sci Rep. 2017;7:4155. 28646181 10.1038/s41598-017-04395-0 PMC5482805

[33] Lin L, Wang H, Ni M, Rui Y, Cheng TY, Cheng CK, et al. J Orthop Transl. 2014;2:35–42.

[34] Al Mustafa M, Agis H, Müller HD, Watzek G, Gruber R. Clin Oral Implants Res. 2015;26:15–9. 24372935 10.1111/clr.12294

[35] Dong W, Zhang T, Epstein J, Cooney L, Wang H, Li Y, et al. Chem Mater. 2007;19:4454–9.

[36] Diu T, Faruqui N, Sjöström T, Lamarre B, Jenkinson HF, Su B, et al. Sci Rep. 2014;4:7122. 25409910 10.1038/srep07122 PMC4238011

[37] Bright R, Hayles A, Wood J, Ninan N, Palms D, Visalakshan RM, et al. Nanomaterials. 2022;12:1140. 35407257 10.3390/nano12071140 PMC9000892

[38] Huang Y-Z, He S-K, Guo Z-J, Pi JK, Deng L, Dong L, et al. Mater Sci Eng: C. 2019;94:1–10. 10.1016/j.msec.2018.08.069 30423681

[39] Feist TP, Davies PK. J solid state Chem. 1992;101:275–95.

[40] Suzuki Y, Pavasupree S, Yoshikawa S, Kawahata R. J Mater Res. 2005;20:1063–8.

[41] Lavenus S, Pilet P, Guicheux J, Weiss P, Louarn G, Layrolle P. Acta biomaterialia. 2011;7:1525–34. 21199693 10.1016/j.actbio.2010.12.033

[42] Gill P, Musaramthota V, Munroe N, Datye A, Dua R, Haider W, et al. Mater Sci Eng: C. 2015;50:37–44. 10.1016/j.msec.2015.01.009 PMC4388742 25746243

[43] Dua R, Centeno J, Ramaswamy S. J Biomed Mater Res Part B: Appl Biomater. 2014;102:922–32. 10.1002/jbm.b.33073 24259264

[44] Subramaniam M, Jalal SM, Rickard DJ, Harris SA, Bolander ME, Spelsberg TC. J Cell Biochem. 2002;87:9–15. 12210717 10.1002/jcb.10259

[45] Harris SA, Enger RJ, Riggs LB, Spelsberg TC. J Bone Miner Res. 1995;10:178–86. 7754797 10.1002/jbmr.5650100203

[46] Brugmans M, Cassiman JJ, Vanderheydt L, Oosterlinck AJ, Vlietinck R, Van Den Berghe H. Cytometry: J Int Soc Anal Cytol. 1983;3:262–8. 10.1002/cyto.990030406 6185285

[47] Gill P, Munroe N, Dua R, Ramaswamy S. J Biomater Nanobiotechnol. 2011;3:10–3.

[48] Lordeus M, Estrada A, Stewart D, et al. Journal of Long-Term Effects of Medical Implants. 2015;25:1–2. 10.1615/jlongtermeffmedimplants.2015011716 25955009

[49] Dua R, Sharufa O, Terry J, et al. Front Bioeng Biotechnol. 2023;11:1202499. 10.3389/fbioe.2023.1202499 PMC10517429 37744253

[50] Cheng CT, To S. Fly Cutting Technology for Ultra-precision Machining. 2023;393–412.

[51] Lee N, Park J, Miralami R, Yu F, Skaines N, Armstrong M, et al. Biomimetics. 2022;7:91. 35892361 10.3390/biomimetics7030091 PMC9326640

[52] Anitha V, Banerjee AN, Joo SW, Min BK. Nanotechnology. 2015;26:355705. 26246034 10.1088/0957-4484/26/35/355705

[53] Jaggessar A, Tesfamicheal T, Wang H, Yan C, Yarlagadda PK. Procedia Manuf. 2019;30:373–9.

[54] Lu CL, Lin YH, Wong WW, Lin HH, Ho MW, Wang NC, et al. Chem Phys Lett. 2011;508:258–64.

[55] Al-Azzam N, Alazzam A. Plos one. 2022;17:e0269914. 35709175 10.1371/journal.pone.0269914 PMC9202894

[56] Arima Y, Iwata H. Biomaterials. 2007;28:3074–82. 17428532 10.1016/j.biomaterials.2007.03.013

[57] Wei J, Igarashi T, Okumori N, Igarashi T, Maetani T, Liu B, et al. Biomed Mater. 2009;4:045002. 19525576 10.1088/1748-6041/4/4/045002

[58] Majhy B, Priyadarshini P, Sen A. RSC Adv. 2021;11:15467–76. 35424027 10.1039/d1ra02402g PMC8698786

[59] Li Y, Xiong J, Wong CS, Hodgson PD, Wen Ce. Tissue Engineering Part A. 2009;15:3151–9. 10.1089/ten.TEA.2009.0150 19351266

[60] Zareidoost A, Yousefpour M, Ghaseme B, Amanzadeh A. J Mater Sci: Mater Med. 2012;23:1479–88. 22460230 10.1007/s10856-012-4611-9 PMC3368253

[61] Zhang Y, Chen SE, Shao J, van den Beucken JJ. ACS Appl Mater interfaces. 2018;10:36652–63. 30270615 10.1021/acsami.8b10992 PMC6213029

[62] Sola-Ruiz MF, Perez-Martinez C, Labaig-Rueda C, Carda C, Martín De Llano JJ. Int J Mol Sci. 2017;18:823. 28406458 10.3390/ijms18040823 PMC5412407

[63] Levin M, Spiro RC, Jain H, Falk MM. Med Devices: Evid Res. 2022;15:103–19. 10.2147/MDER.S360297 PMC9056099 35502265

[64] Wang C, Gao S, Lu R, Wang X, Chen S. Cells. 2022;11:3417. 36359812 10.3390/cells11213417 PMC9657283

[65] Piszczek P, Radtke A, Ehlert M, Jędrzejewski T, Sznarkowska A, Sadowska B, et al. J Clin Med. 2020;9:342. 31991841 10.3390/jcm9020342 PMC7073575

[66] Przekora A. Mater Sci Eng: C. 2019;97:1036–51. 10.1016/j.msec.2019.01.061 30678895

